# Physical properties of computationally informed phyto-engineered 2-D nanoscaled hydronium jarosite

**DOI:** 10.1038/s41598-022-25723-z

**Published:** 2023-02-10

**Authors:** N. L. Botha, K. J. Cloete, G. G. Welegergs, M. Akbari, R. Morad, L. Kotsedi, N. Matinise, R. Bucher, S. Azizi, M. Maaza

**Affiliations:** 1 UNESCO-UNISA Africa Chair in Nanosciences and Nanotechnology Laboratories, College of Graduate Studies, University of South Africa, Muckleneuk Ridge, P. O. Box 392, Pretoria, 0003 South Africa; 2 Nanosciences African Network (NANOAFNET), iThemba LABS-National Research Foundation, P. O. Box 722, Somerset West, 7129 Western Cape South Africa; 3 Department of Chemistry, College of Natural Science, Debre Berhan University, P. O. Box 445, Debre Birhan, Ethiopia

**Keywords:** Chemistry, Nanoscience and technology

## Abstract

This study describes a molecular dynamics computational modelling informed bioengineering of nano-scaled 2-D hydronium jarosite. More specifically, a phyto-engineering approach using green nano-chemistry and agro-waste in the form of avocado seed natural extract was utilized as a green, economic, and eco-friendly approach to synthesize this unique mineral at the nanoscale via the reduction of iron (II) sulphate heptahydrate. The nanoproduct which was found to exhibit a quasi-2D structure was characterized using a multi-technique approach to describe its morphological, optical, electrochemical, and magnetic properties. Radial distribution function and electrostatic potential maps revealed that flavone, a phenolic compound within the avocado seed natural extract, has a higher affinity of interaction with the nanoparticle's surface, whilst vanillic acid has a higher wetting tendency and thus a lower affinity for interacting with the hydronium jarosite nanoparticle surface compared to other phytoactive compounds. XRD and HRTEM results indicated that the nanoscale product was representative of crystalline rhombohedral hydronium jarosite in the form of quasi-triangular nanosheets decorated on the edges with nanoparticles of approximately 5.4 nm diameter that exhibited significant electrochemical and electroconductive behaviours. Magnetic studies further showed a diamagnetic behaviour based on the relationship of the inverse susceptibility of the nanomaterial with temperature sweep.

## Introduction

In 2004 the Opportunity rover reported widespread existence of jarosite at Meridiani Planum 2^1^, Since then, the mineral has been frequently identified on Mars^2–4^ and has been interpreted as an evidence for the occurrence of liquid water^5^ as on Earth, jarosite forms as the result of acidic oxidative low-temperature weathering of iron-bearing minerals in water-limited settings^6^. Likewise, jarosite crystals adhering on residual silica-rich particles have been identified in the Talos Dome ice core (East Antarctica) and interpreted as products of weathering involving aeolian dust and acidic atmospheric aerosols^7,8^.

**Figure 1:**
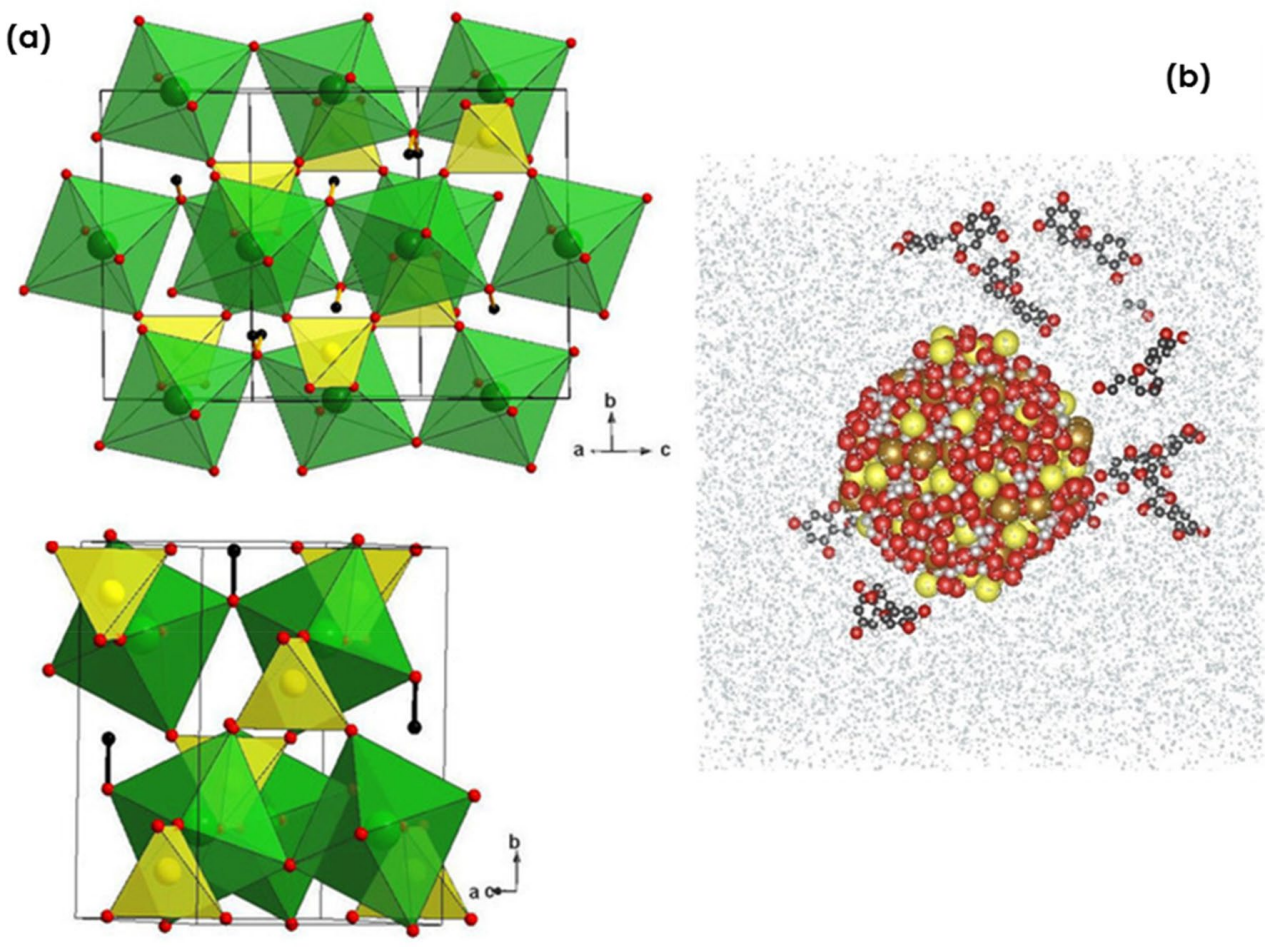
(**a**) Typical layered crystallographic structure of Jarosite, (**b**) Schematic view of a hydronium jarosite nanoparticle surrounded by 10 molecules of Epicatechin and water molecules in a simulation box.

Hydronium jarosite is a relatively rare mineral belonging to the jarosite series of secondary iron sulfate minerals synthesized via the oxidation of sulfide minerals, in particular, pyrite^9^. As examplified by Fig. 1a, Jarosites are layered systems characterized by the formula A_1__−x_(H_3_O)xFe_3_ + 3-y(SO_4_)_2_(OH)_6_-3y (H_2_O)3y, where the A sites are occupied by a monovalent (K, Na, H_3_O^+^, NH_4_, Ag and Tl) or divalent (Pb) cation^10^, with hydronium jarosite [H_3_OFe_3_ (SO_4_)_2_(OH)_6_] containing an elusive H3O^+^ group that was previously characterized in more detail by Majzlan et al.^11^. Jarosite-type minerals generally occur in fluvial environments contaminated by acid rock or acid mine drainage sediments or in mine tailings of sulfide ore deposits. They are of economic importance, and show potential for utilization in multifunctional applications^12^. For example, hydronium jarosite serves as an iron scavenger in the hydro-metallurgic industry as a raw material in the production of pigments or construction materials^13–18^, as a heterogeneous Fenton catalyst in the degradation of azo dye methyl orange (MO)^19^, and for the photocatalytic reduction of specific carcinogenic and mutagenic organic as well as inorganic pollutants such as Cr(VI)^20^ released by leather tanning, metal plating, pigment manufacturing, and chromate production industrial activities among others.

Since hydronium jarosite minerals harbour diverse catalytic applications within the chemical industry and as an effective photocatalytic reducing agent with potential applications in environmental remediation, researchers have tried to synthesize such jarosite-type minerals using both chemical and biological oxidation of Fe approaches^21,22^. In this regard, Jarosite minerals have been synthesized using different methods including chemical synthesis^23^. Even though some jarosite minerals only require Fe_2_ (SO_4_)_3_ and water, some also require the use of high temperatures and necessitate longer synthesis times^19^. Chemical methods employ a number of toxic chemicals^24^, whilst the biological oxidation of Fe during hydronium jarosite formation is significantly affected by acidity and temperature^25,26^. Only very recently, the synthesis and characterization of rod-shaped jarosite nanoparticles using a microwave-assisted hydrothermal method have also been reported^25^. As nanomaterials have been shown to have increased reactivity for application across multidisciplinary research fields due to their unique physico-chemical properties and larger surface to volume ratio, the catalytic potential of hydronium jarosite may be further developed by tuning its physico-chemical properties to the nanoscale. Yet, there is no literature available on the engineering of hydronium jarosite at the nanoscale.

Phyto-engineering of nanomaterials has increased in popularity over recent years due to the fact that this approach is environmentally safe and possible at standard conditions of temperature and pressure^26^. More specifically, phyto-engineering involves the use of plant extracts to produce safe and biocompatible nanomaterials^27^. Plants contain phytochemicals including phenolic compounds that have been shown to serve as excellent reducing and chelating agents for synthesizing nanomaterials^27,28^. Since the interaction between different types of plant phytochemical compounds and nanoparticles plays a critical role in phyto-engineering an end product with unique physico-chemical properties^27^, delineating this interaction at the molecular level is important. Yet, studies exploring the molecular interaction between specific plant phytochemical compounds and nanoparticles using methods such as computational simulation remain to our knowledge largely limited. In effect, molecular dynamics computational simulation—a technique for exploring conformational space using various sized molecules, including proteins, active compounds, and nanoparticles^29–31^—may present as a powerful computational approach to explore the interaction between plant phytochemical compounds serving as nanoparticle stabilizing agents during phyto-engineering of nanomaterials that may exhibit unique physico-chemical properties for diverse applications.

This study reports for the first time on the synthesis of nano-scaled 2-D Hydronium jarosite. More precisely, this study combines computational simulation for informing the phyto-engineering of hydronium jarosite nanoparticles utilizing an agro-waste product rich in phytochemicals—*Persea americana* (avocado) seeds^32^—as reducing and chelating agents for the reduction of iron (II) sulphate hepta-hydrate (FeSO_4_·7H_2_O) using green nano-chemistry approach. More specifically, the interactions between hydronium jarosite nanoparticles, water, and eight phyto-active, including phenolic compounds, found in avocado seeds (caffeic acid, chlorogenic acid, epicatechin, ferulic acid, flavone, neochlorogenic acid, procyanidin, and vanillic acid) were investigated using a molecular dynamics computational simulation. In addition, full physicochemical characterization of the hydronium jarosite phyto-engineered at the nanoscale were studied using a multi-technique approach. Although hydronium jarosite minerals have a unique chemical formulation that may endow it with prominent optical, electrical, electrochemical, and magnetic features not yet explored, this study also investigated these parameters in phyto-engineered hydronium jarosite nanoparticles.

## Materials and methods

### Chemicals used for nanoparticle synthesis

Analytical grade iron (II) sulphate hepta-hydrate (FeSO_4_·7H_2_O) and Alconox were purchased from Sigma-Aldrich. The glassware used in all experiments was washed with Alconox and rinsed thrice with DI H_2_O. All aqueous solutions were prepared with double distilled deionized (DI) H_2_O.

#### Preparation of avocado seed aqueous extract and hydronium jarosite nanoparticles

The preparation of avocado seed aqueous extract and phyto-engineering of hydronium jarosite nanoparticles were completed by following a green chemistry approach and the method of Bhattacharjee et al.^33^. The mixture constituted of FeSO_4_·7H_2_O dissolved in DI water and combined with avocado seed extract was stirred at 70 °C for 3 h. The hydronium jarosite nanoparticles were separated and isolated from the solution using centrifugation at 4000 rpm for 20 min. The pellet obtained after centrifugation was washed with DI H_2_O and dried in a Memmert hot air oven at 50 °C for 2 h.

### Morphological, elemental, optical, electrochemical and magnetic characterization

Sample purity and elemental composition was determined with Energy Dispersive X-rays Spectroscopy (EDS) using a Nova Nano-SEM equipped with an Oxford X-Max detector (20 mm^2^) at 20 kV and a working distance of 6 mm and Oxford INCA software. HRTEM and SAED images were captured using a Tecnai F20 FEG-TEM at 200 keV in bright field mode. The Direct Electron DE16 camera detection system was used to capture images, whilst ImageJ open source software was used to evaluate particle size distribution.

Phase identification and sample crystallography were performed using X-Rays Diffraction (XRD) measured with a Bruker Advanced AXS D8 diffractometer with a monochromatic Cu_K-1_ radiation (λ = 1.54060 Å) operating in the Bragg–Brentano geometry and a step size of 0.2° with 0.2 s per step for angles between 5° and 85°. To characterize chemical bonds and the molecular structure of functional groups originating from organic phyto-constituent molecules responsible for the reduction and stabilization of the Fe ions, Fourier Transform Infrared Spectroscopy (FTIR) using potassium bromide pellet technology and a PerkinElmer 100 Spectrometer in the wave number 400–4000 cm^−1^ range was used.

For the optical characterization, UV–VIS absorbance and photoluminescence investigations were carried out. A Cary5000 UV–VIS–NIR spectrophotometer with double beam and an integrating sphere was used to measure UV–VIS absorbance at room temperature within the spectral range of 200–800 nm for the avocado seed and colloidal nanoparticle solution and hence the bio-reduction and formation of the nanoparticles. A fibre-optics linked ocean optics system consisting of a high-powered UV Light emitting diode source (240 nm) coupled to a high sensitivity QE ProFL spectrometer was used for the photoluminescence measurements.

For the magnetic characterization, a cryogenics vibrating stage magnetometer was used to study the magnetic properties of the sample. The sample was mounted on a sample holder and centred between two pick up coils, while the sample stage vibrated at a frequency of 50 Hz and at an approximate amplitude range of 3 mm. The zero field cooling (ZFC) and field cooling (FC) measurements were conducted from 3 to 300 K at a set field of 1 T.

For the electrochemical investigations, electrochemical properties of the material were conducted on a CH Instruments Autolab Potentiostat electrochemical workstation using two techniques including cyclic voltammetry (CV) and electrochemical impedance spectroscopy (EIS). The instrument is a three-electrode system in which a glassy carbon electrode is used as a working electrode, a platinum wire as a counter electrode, and Ag/AgCl as reference electrodes combined with a 3 M NaCl salt bridge solution. CV experiments were performed on the potential window from − 0.6 to 0.6 V with a scanning rate of 50 mV/s and various scans from 20 to 100 mV/s. EIS measurements were conducted at a perturbation amplitude of 10 mV within the frequency range of 0.1–100 Hz. All experimental measurements were conducted using a 0.1 M KOH solution as an electrolyte. High purity argon gas was used to de-oxygenate and blanket the experimental solution during all measurements at room temperature. The working electrode (GCE/hydronium jarosite) was prepared by adding 2 µl of a 5% Nafion solution in a small amount of hydronium jarosite material dissolved in ethanol. The mixture was ultra-sonicated in a warm water bath for 15 min to make a colloidal solution. For the analysis, a small amount of the solution was drop-coated on the surface area of the glassy carbon electrode that was cleaned before coating using alumina powder (1.0, 3.0, 0.05) followed by ultra-sonication in ethanol and subsequently water, for 5 min. The modified glassy carbon electrode was dried for 20 min in an oven at 35 °C before being immersed into 0.1 M KOH for the experimental measurements.

### Computational and modelling: molecular dynamics computational simulation

To perform the molecular dynamics simulations, a sphere of hydronium jarosite nanoparticles produced by Atomsk^34^ with a diameter of 2.5 nm and containing 981 atoms were placed in the centre of a simulation cubic box. Ten molecules of each phyto-active compound were subsequently randomly inserted into the box, with a minimum distance of 1 nm from the simulation box's edge sides (Fig. 1). GROMACS 2019 software^35^ was used to perform all molecular dynamics simulations under periodic boundary conditions, using the CHARMM36 force field^36^ and SPC water model^37^. The TIP3P model was used to solvate the water molecules in the complex. The systems were optimized in terms of energy using the steepest descent algorithm for all atoms^38^. Each system was equilibrated using the NVT ensemble [constant number of particles (N), volume (V), and temperature (T)] coupled to the V-rescale thermal bath at 300 K for 200 ps, and the NPT ensemble (constant number of N, P, and T) coupled to the Berendsen pressure bath at 1 atm for 300 ps. Each system was then conducted at a 50 ns molecular dynamics simulation with a time step of 2 fs under constant conditions of 1 atm, and 300 K. The LINCS algorithm^39^ was used to constrain the lengths of H-bonds. The particle mesh Ewald^40^ approach was used to apply long-range electrostatics. GROMACS utilities were used to analyze the trajectory data and VMD 1.9.3^41^ was used to produce molecular graphics and visualizations. The force fields of hydronium jarosite nanoparticles and compounds were determined using CHARMM CGenFF^42^. The electronic structures of eight phytoactive chemicals (caffeic acid, chlorogenic acid, epicatechin, ferulic acid, flavone, neochlorogenic acid, procyanidin, and vanillic acid) were done at the density functional theory (DFT) level using the Gaussian program, version 09^43^. The geometry optimization of the molecules was carried out at the B3LYP/6-311++g(d,p) level of theory.

### Ethical statement

In the study, avocado seeds were used and this requires no compliance with any institutional, national, and international guidelines and legislation since the avocado seeds presented as household waste in the form of food waste.

## Results and discussion

### Morphology and size distribution studies

**Figure 2:**
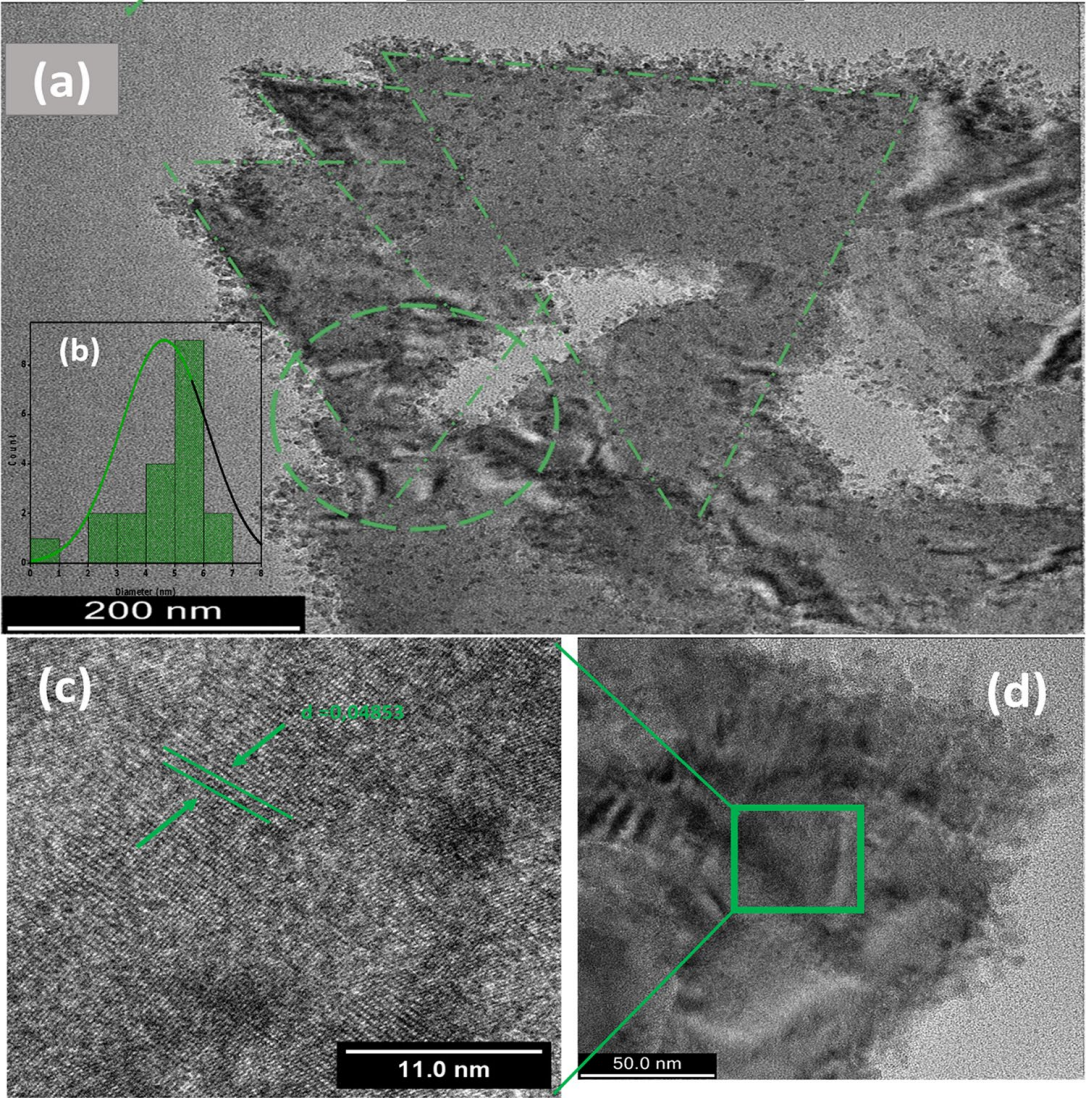

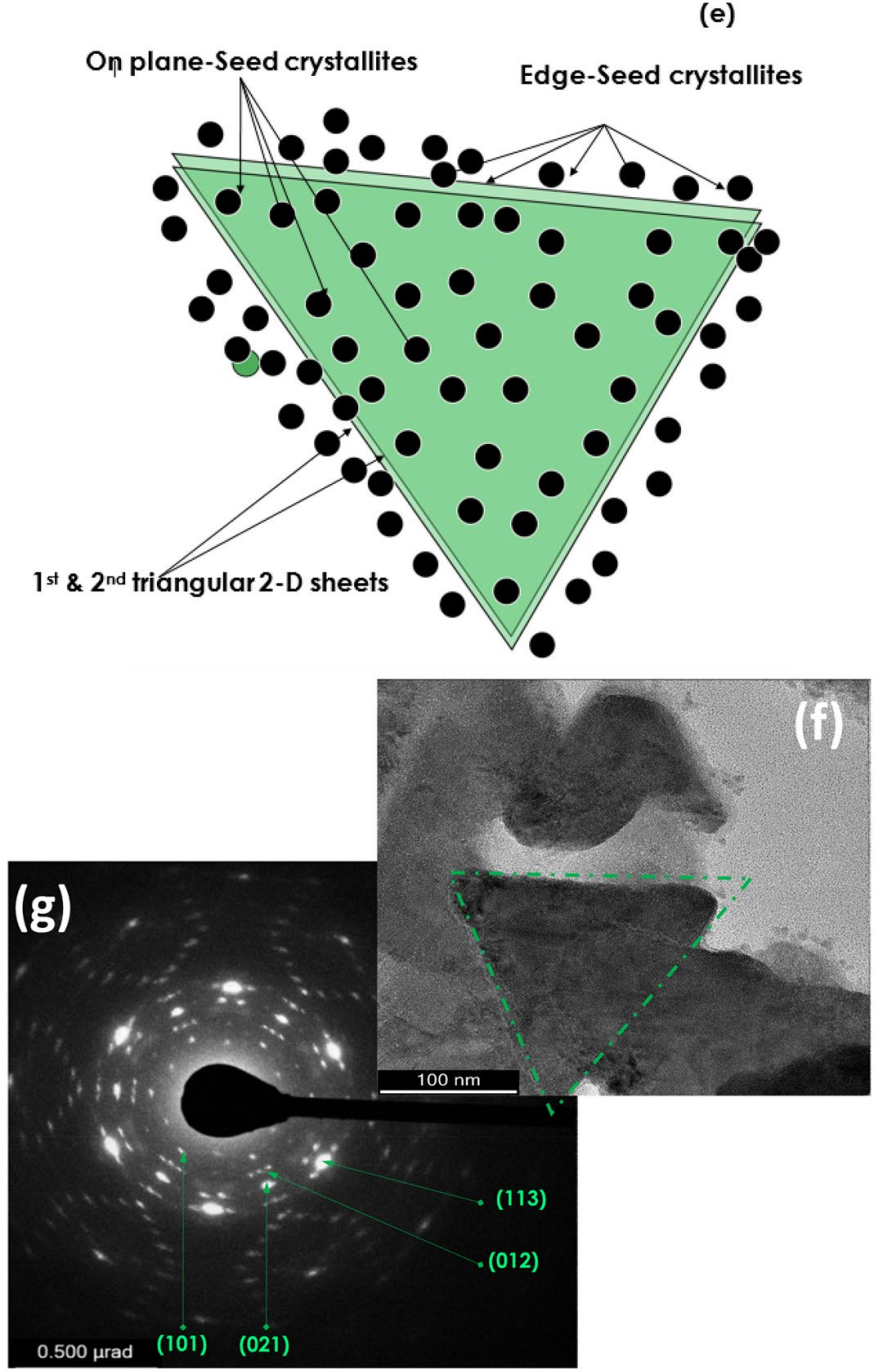
HRTEM images of the prepared hydronium jarosite nanoparticles. (**a**) Shows the2-D sheets decorated by nanoparticles, (**b**) particle size distribution histogram, (**c**,**d**) shows the lattice fringe zoomed out, (**e**) summary diagram of the (**a**), (**f**) shows the thick triangular sheet, (**g**) SAED image.

The shape and the size of the green synthesized hydronium jarosite particles were characterized by high resolution transmission electron microscopy (HRTEM). Figure 2 displays a typical HRTEM and SAED images and size distribution histogram of the hydronium jarosite nanoparticles. The HRTEM image of Fig. 2a indicates that the bio-engineered material exhibits a crystal clear shape anisotropy. More precisely, they seem forming triangular shaped 2-D sheets grafted with nanoparticles with a significant nano-particles population both on the basal surface and at the edge of the 2-D triangular sheets as schematically summarized in Fig. 2e. The measured average length of the triangular shaped sheets shown in is ~ 193.03 nm. The agglomeration of the particles at the edges of the sheets may be due to the electrostatic field on the edges as their chemical environments are different from the atoms on the basal plane due to the termination of these atoms^44^. The average diameter of the decorating particles is ~ 5.4 nm. The histogram diagram of their particle size distribution is displayed in Fig. 2b.

Yet it is early to suggest the growth model of the obtained triangular 2-D nanosheets, it is likely that the process of growth follows the summarized model of Fig. 2c. If one borrows from the thin films epitaxial growth governed by the 3 major models; Volmer–Weber, Stranski–Krastanov and Frank–van der Merwe^45,46^, it is likely the last one that fits with the current observations. If so, within this model, at room temperature, through the Brownian motion, their kinetic energy can be approximated as 3/2k_B_T ~ 4.23 10^–2^ eV, the so called on plane-crystallites diffuse (equivalent of seed atoms/clusters) to coalesce so to form the 2-D sheets controlling the thickness of the formed sheets. The edge crystallites are likely to diffuse reaching the edge of the 2-D sheets contributing to their spatial extension. If so, the 2-D sheets growth would be similar to that of thin films governed by Frank–van der Merwe model^47^. If so, the current jarosite growth would be similar to that observed on graphene by Wang et al.^48^. Also, this growth is common to to the formation mechanism in a series of antiprismatic jarosite-type compounds as validated by Xin Yang et al.^49^, Fig. 2c and its zoom Fig. 2d display parallel lattice fringes with an inter-reticular d- spacing of to 0.48 nm as measured by Image-J suggesting that the 2-D sheets present a certain degree of crystallinity at least locally. The Selected Area Electron Diffraction (SAED) (Fig. 2g) on a thicker jarosite 2-D sheet of Fig. 2f suggests that the prepared Jarosite 2-D sheets are crystalline in nature with a significant textured orientation (highly intense/bright spots) with a likely hexagonal crystallographic symmetry which is an intrinsic crystallographic orientation of hydronium jarosites^50^.

### Crystallographic properties

**Figure 3:**
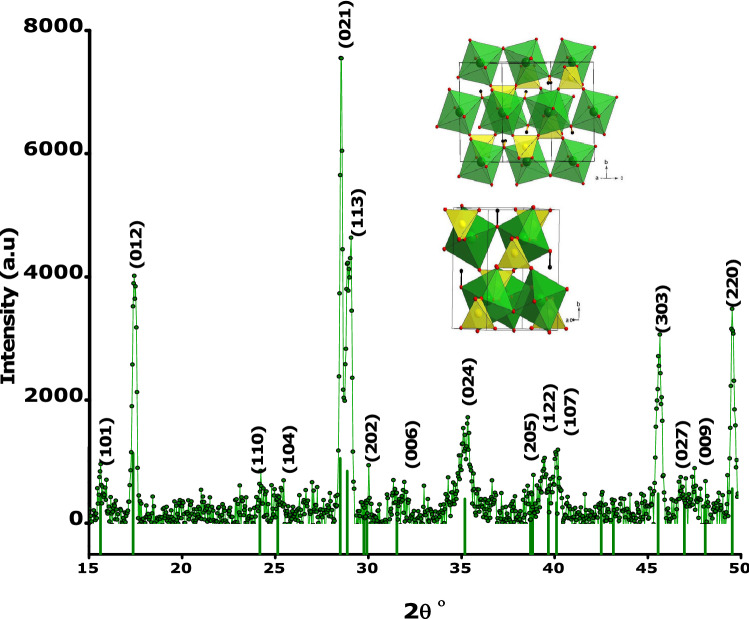
X-ray diffraction in comparison with the standard JCP2_31-0650 card data.

In relation to the crystallographic properties of the bio-engineered sample, Fig. 3 reports the room temperature X-Rays Diffraction pattern (XRD) of the bio-engineered Hydronium jarosite nanoparticles. The spectrum displays a rich set of Bragg diffraction peaks located at 14.8°, 15.6°,17.3°, 24.1°,25.1°, 28.4°, 28.8°, 29.9°, 31.5°, 35.1°, 38.8°, 39.6°, 45.5°, 46.9°, 48.0° and 49.5° corresponding to the (JCP2_31-0650) Bragg peaks of (101), (003), (012), (110), (104), (021), (113), (202), (006), (024), (122), (303), (027), (009) and (220), respectively. Following a Maud and a Rietveld treatment, such an XRD pattern is in full agreement with the rhombohedral hydronium jarosite (JCP2_31-0650)^51,52^. It is worth noting that the XRD patterns showed no impurities or other competitive jarosite phases. These patterns are comparable with the reported hydronium jarosites obtained through bio-mineralization^53^ and hydrothermal synthesis^51^. The XRD peaks further confirm the crystallinity of the nanoparticles, in support of the previous SAED data.

### Vibrational properties

**Figure 4:**
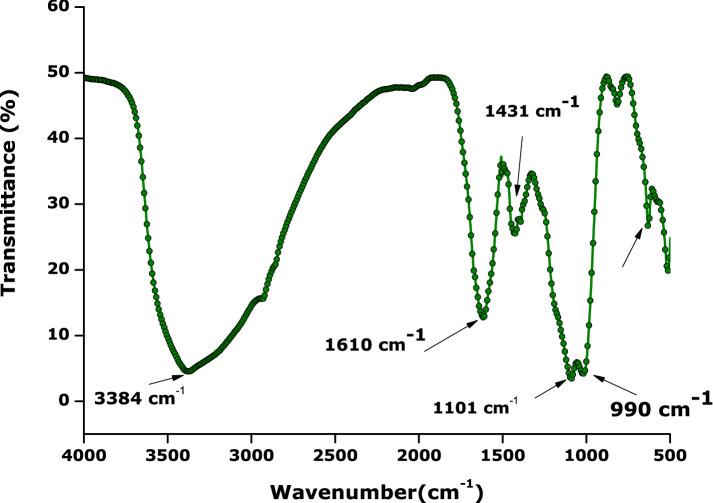
Fourier transform infrared spectra of the prepared hydronium jarosite nanoparticles.

In relation to the atomic vibrational properties of the bio-engineered sample, Fig. 4 displays the FTIR spectrum of the bio-engineered Hydronium jarosite sample. The spectrum has a broad stretching band in the region from 3000 to 3600 cm^−1^ ascribed to the ν(O‒H) vibration from the hydronium ions at 3390 cm^−1^. The vibrations at 1439 and 1392 cm^−1^ are attributed to ν(O‒H) whereas the doublets in the region 1000–1200 cm^−1^ at 1092 cm^−1^ and 1018 cm^−1^ are assigned to SO_4_ vibrations. The spectrum also shows metal coordination bands around 600 to 400 cm^−1^ which are assigned to the FeO_6_ coordination^19,54^. The band at around 1600 which, according to Plasil et al.^10^, is due to the bending modes of H_2_O and H_3_O^+^ that overlap, which is mainly a result of the OH and H_3_O interaction forming H_2_O. In a summary, the FTIR spectrum of the bio-engineered Hydronium jarosite sample exhibits the same stretching bands and vibrations as the various reported jarosite analogues^55^. This confirms that bio-engineered 2-D nanosheets are as a Hydronium jarosite analogue.

### UV–VIS, photoluminescence

**Figure 5:**
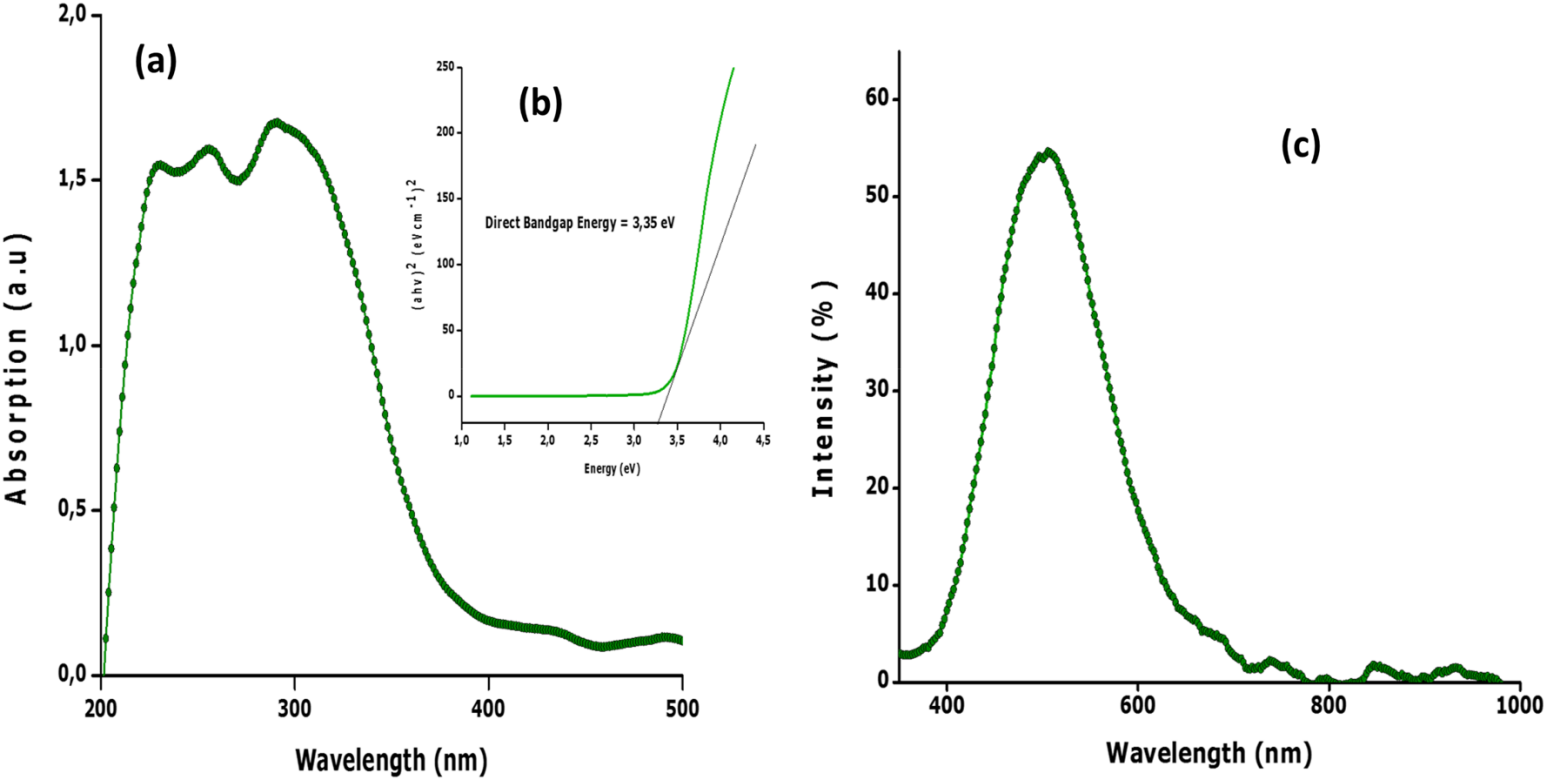
(**a**) UV–VIS spectroscopy, (**b**) Tauc plot, and (**c**) photoluminescence of the prepared hydronium jarosite nanoparticles.

In relation to the optical properties of the bio-engineered 2-D hydronium jarosite sample, Fig. 5 reports the UV–VIS absorbance spectrum (a) and its corresponding Tauc plot (b) as well as the fluorescence spectrum (c), of the bio-engineered 2-D hydronium jarosite sample. The UV–VIS absorbance spectrum (Fig. 5a) displays a relatively broad absorption maxima in the UV region, with two shoulders centered at the vicinity of 242 and 287 nm which might be attributed to the charge transfer between valence and conduction bands^56^. Such an elevated UV-Bleu optical absorbance could be exploited for photocatalytic activity in waste water treatment. The absorbance data was used to estimate the optical band gap by employing the Tauc’s approximation^57^. The derived direct band gap energy is about ~ 3.4 eV. The Photoluminescence data displays several emissions with the maximum centered at the vicinity of 500 nm. While this latter is likely to be caused by surface oxygen deficiencies, the others in the near Infrared region (~ 750 nm, ~ 850 nm, ~ 770 nm, ~ 950 nm) could be ascribed to volume O or Fe defects.

### Elemental chemical properties

**Figure 6:**
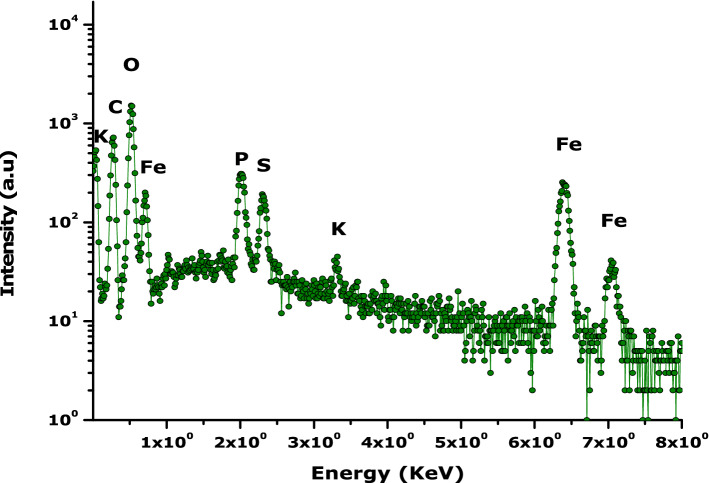
Dispersive energy of the prepared hydronium jarosite nanoparticles.

Figure 6 displays a typical Energy Dispersive X-rays Spectroscopy spectrum of the bio-engineered 2-D hydronium jarosite sample. In addition to the Jarosite major elements of Fe, K and O, one can distinguish Phosphorus at energy channels centered at the vicinity of 2.1 keV. The presence of this contaminant is likely to originate from the avocado seed extract used as a chelating agent. Its small ionic radius is likely to favour their diffusion within the crystallographic open channels offered by the bio-engineered 2-D hydronium jarosite nanoparticles.

### Magnetism properties

**Figure 7:**
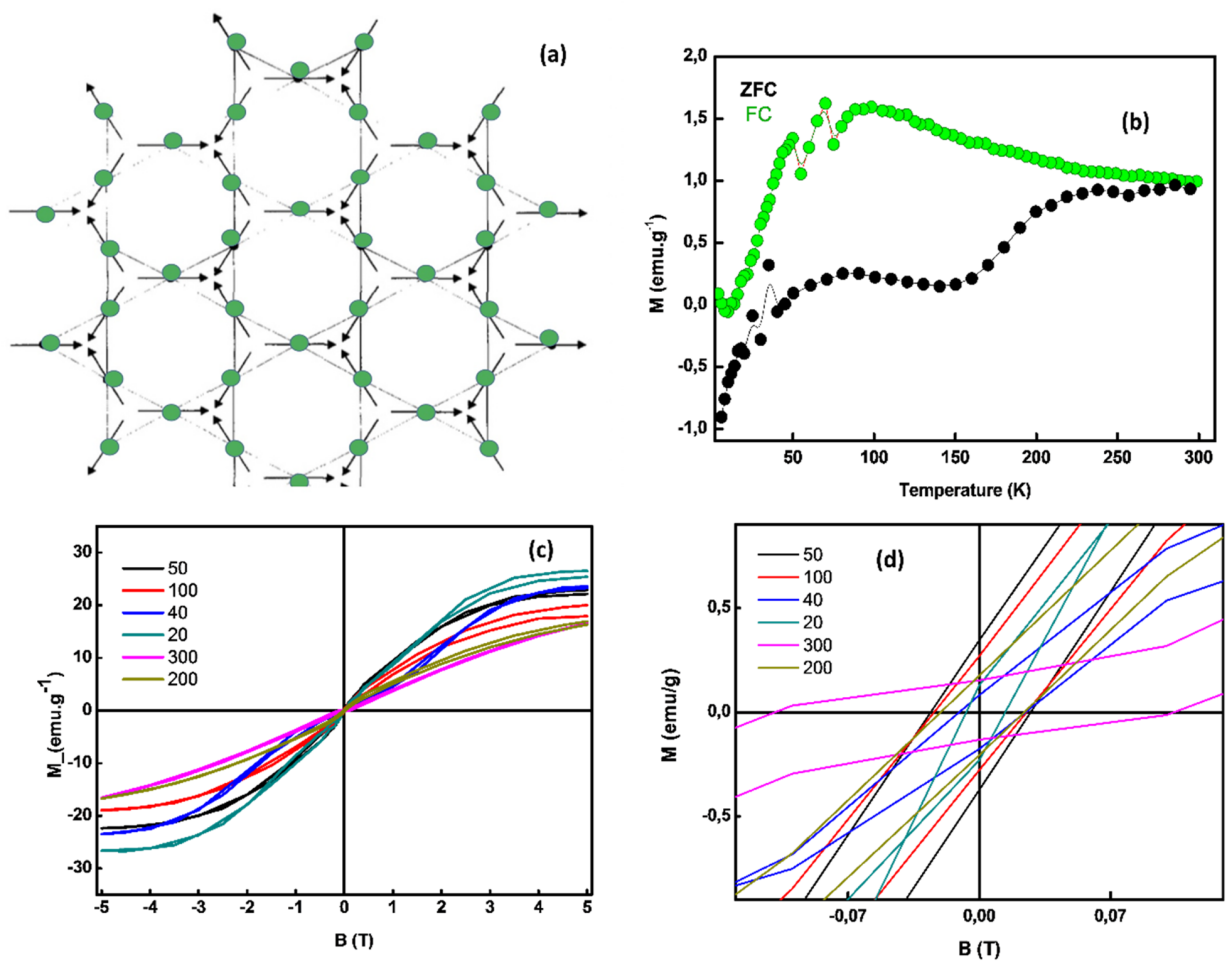
(**a**) Kagome lattice of anti-ferromagnetically coupled Heisenberg spins with the so-called q = 0 structure in Jarosites, (**b**) plot of zero field cool (ZFC) and field cool (FC) of the sample showing a blocking temperature of the sample, (**c**) inverse susceptibility plot versus temperature with the applied field at 1 T. (**d**) Magnetization plot of the sample at various temperatures and (**e**) zoomed in plots of the magnetization of the sample at various temperatures.

As established, the jarosite family provides an ideal model for Kagome antiferromagnets as schematically illustrated in Fig. 7a. The Fe^3+^ ions sit at the vertices of well-separated Kagome layers and behave as an S = 5/2 Heisenberg spins coupled through strong nearest-neighbour antiferromagnetic exchange (Curie–Weiss constants are typically ~ 700 K). Most members of the jarosite family show long-range magnetic order with the q = 0 spin configuration below 55 K as validated by Towsend et al.^58,59^. Likewise, it is to be noted that some jarosites show long-range magnetic order implies that further-neighbour interactions may be significant in these materials, stabilising a particular spin configuration relative to the others^60^. Elucidation of the response of hydronium jarosite to diamagnetic dilution may require a better understanding of further-neighbour exchange in the jarosite family, and of the influence this has on theoretical models. In addition, it has been reported that the jarosite phases that order magnetically at low temperatures tend to behave different from the known magnetic solids^61^. Grohol et al. found KGa_3_(SO_4_)_2_(OH)_6_ to be a diamagnetic analogue of KFe_3_(SO_4_)_2_(OH)_6_ jarosite^62^.

In relation to the magnetic properties of the bio-engineered 2-D hydronium jarosite sample it can be seen in Fig. 7b from the ZFC and FC plots that the two plots intersect at approximately ~ 274 K, which is the region of the blocking temperature of the system. In this region, the spins are random and magnetic domains in the material are not aligned due to thermal component. Figure 7c displays the magnetization curves (from − 5 to 5 T) of the sample measured at the temperatures 20 K, 40 K, 50 K, 100 K, 200 K and 300 K. Zooming in at the axis intercepts of the plots, coercivity, remnant field, and magnetic saturation can be measured as shown in Fig. 7d, which shows that the sample exhibit higher coercivity of 0.11 T at 300 K, with a low coercivity of 0.007 T at 20 K. The remnants field is higher at 50 K. The sample show some saturation magnetization at lower temperatures (20–100 K). While these rich magnetic results are preliminary, it is intended to perform similar studies with SQUID on a single thin and thick triangular sheets of the bio-engineered 2-D hydronium jarosite.

### Electrochemical properties

**Figure 8:**
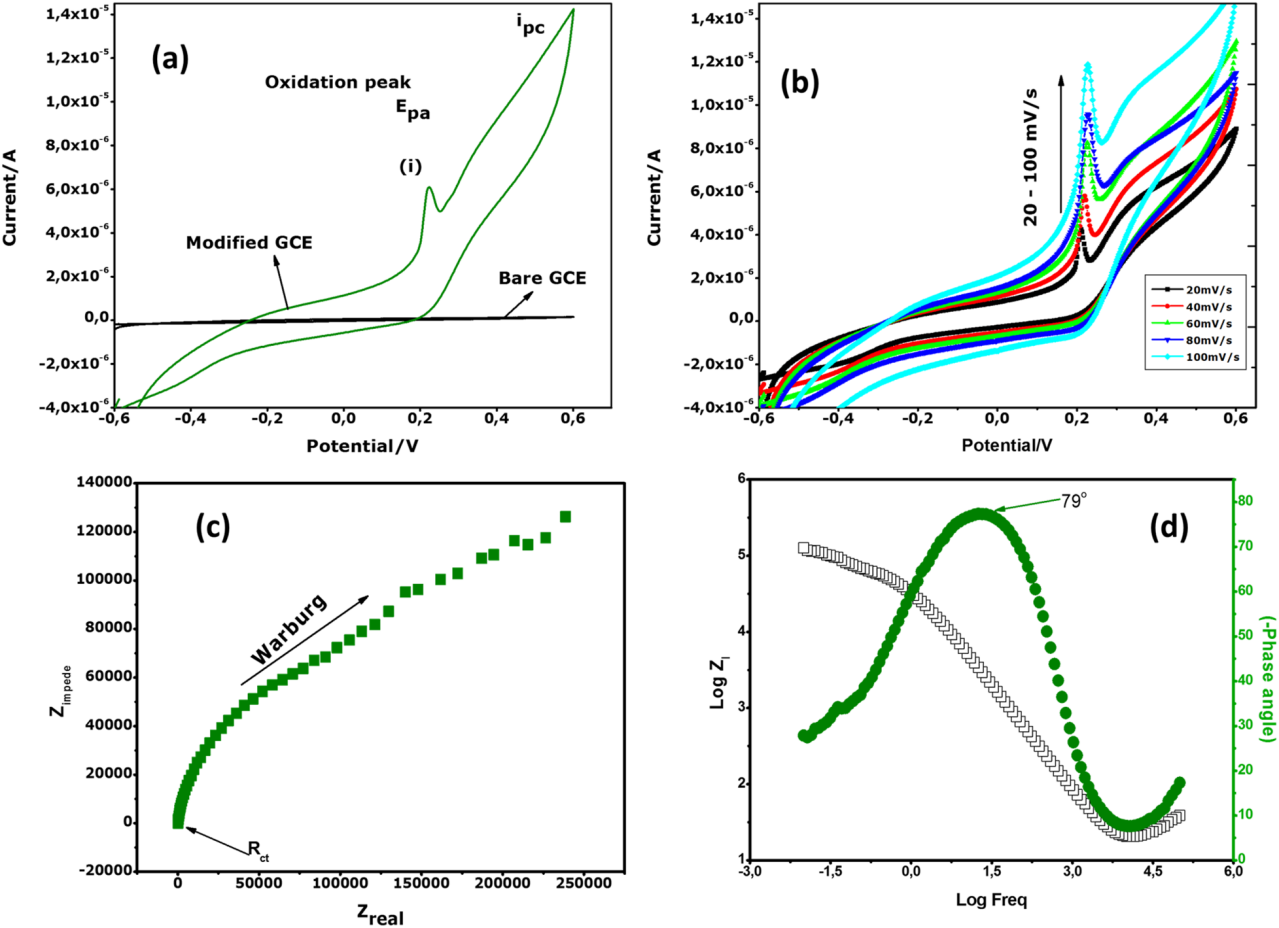
Cyclic voltammetry (CV) curves at 50 mV/s (**a**), CV at various scan rates ranging from 10 to 60 mV/s in 0.1 M NaOH (**b**), Nyquist (**c**), and Bode plot (**d**).

In relation to the electrochemical properties of the bio-engineered sample, cyclic voltammetry is used^63,64^. Figure 8a displays typical voltammograms for bare GCE and deposition of the hydronium jarosite 2-D nanosheets at a scan rate of 50 mV/s and various scan rates from 20 to 100 mV/s. Based on these results, there is no peak observed for the unmodified electrode (bare GCE), with peaks and higher current density observed after the modified electrode with hydronium jarosite nanoparticles. The peaks observed at 0, 22 V and 5, 9 × 10^–6^ represent the strong anodic peak which corresponds to the oxidation process of Fe(II) ions to Fe(III). No clear reduction peak was observed with respect to the potential range, indicating that the oxidation process is pseudo-irreversible. The CV results are an indication that the material have a good electrochemical behaviour, therefore it is considered as a promising electro catalyst for electrochemical applications. The scan rate (v) effect was studied as shown in Fig. 8b, where the chosen v was 20, 40, 60, 80 and 100 mV/s. The performance was evaluated by observing the peak current in the anodic scan. The oxidation peak current increases linearly as a function of scan rate, suggesting a fast diffusion-controlled electrolyte ion transport kinetic at the interface of nanoparticles on the electrode. The oxidation peak potentials shift slightly to more positive values, which may be attributed to the fast faradaic redox reaction because of the good interaction between the conductive GC electrode and the electroactive hydronium jarosite in the alkaline electrolyte.

EIS was used to further investigate the electric conductivity and kinetics of the hydronium jarosite behaviour using the Nyquist and bode plot analysis. Figure 8c shows the Nyquist plot (imaginary Z″ VS real Zʹ) which is a flat semi-circle region observed at the high frequency region ascribed to the low charge transfer resistance Rct or high electric conductivity of the sample. The semi-circle continued by a straight line in a low frequency region, which is related to Warburg impedance or diffusive resistance between the electrode pores and the electrolyte ions. This could be indicating that the electrochemical performance is highly related to the interfacial charge transfer process and diffusion control^65–67^. Figure 8d provides information on the impedance and phase angle as a function of frequency. The bode analysis is plotted log frequency vs log imaginary Zʺ and phase angle (degrees). The phase angle in a plot shows the maximum angle at 79°, this resultant of a higher metallic conductivity of the sample. The results reveal good electronic conductivity as well as electrochemical stability of the hydronium jarosite nanomaterial.

### Molecular dynamics computational simulation

**Figure 9:**
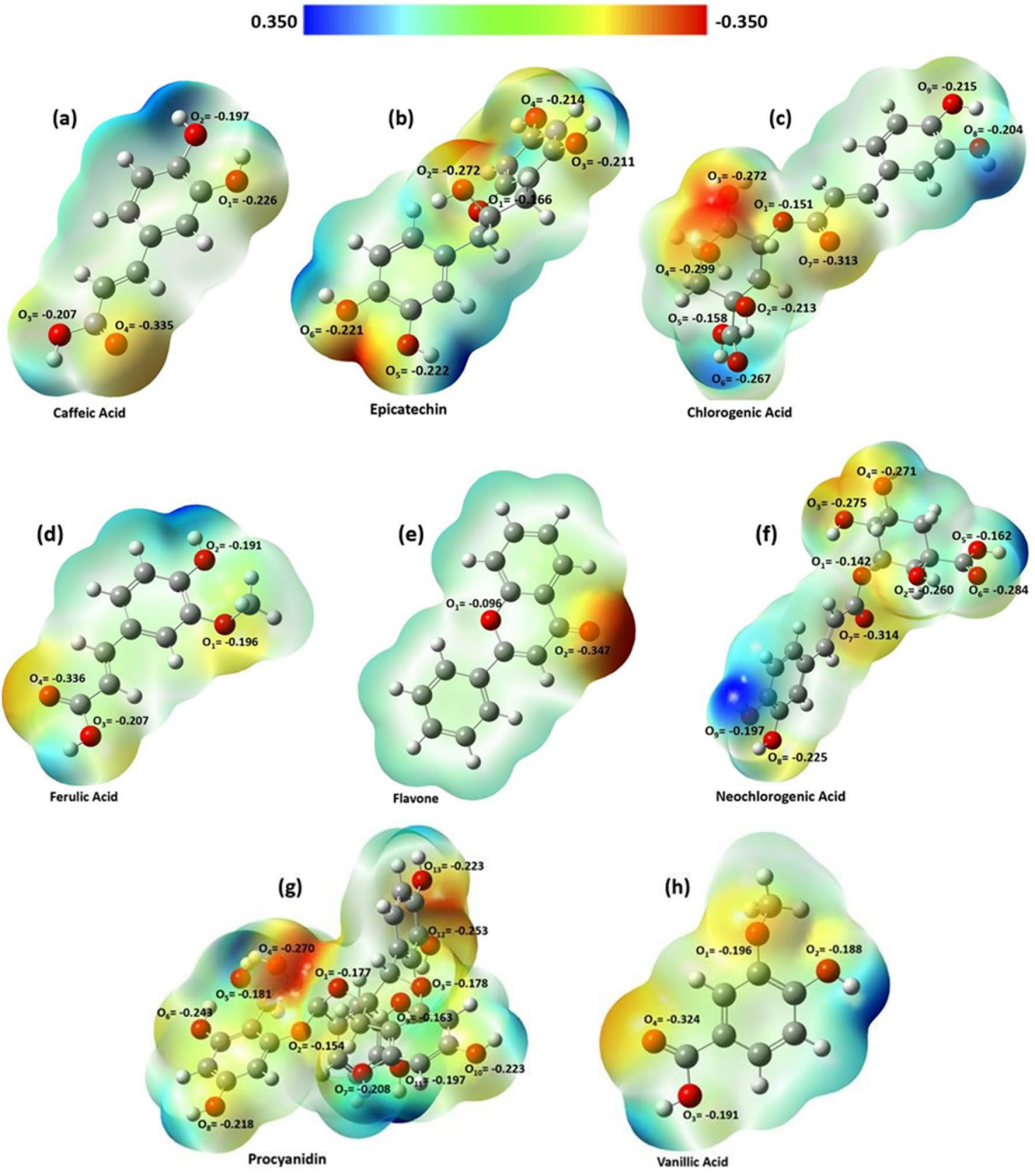
Charge distribution (Hirshfeld point charges) of eight phytoactive compounds and their electrostatic potential map for (**a**) Caffeic acid, (**b**) Epicatechin, (**c**) Chlorogenic acid, (**d**) Ferulic acid, (**e**) Flavone, (**f**) Neochlorogenic acid, (**g**) Procyanidin, and (**h**) Vanillic acid. The scale bar at the top of this figure demonstrates regions of electron excess (reddish area) to regions of electron deficiency (bluish area). The geometry optimization of active compounds was carried out at the B3LYP/6-311++g(d,p) level of theory.

The root mean square displacements (RMSDs) of phyto-active compounds describe when the analysed systems attain their equilibrium states during simulations. Their plots demonstrated that in simulations with drastically reduced RMSD fluctuations, all systems reach their equilibrium state before the duration of 10 ns. As a result, we used the molecular dynamics trajectories extracted after 10 ns for further analysis. The charge distribution and electrostatic potential map of eight active compounds (caffeic acid, chlorogenic acid, epicatechin, ferulic acid, flavone, neochlorogenic acid, procyanidin, and vanillic acid) in Fig. 9 illustrate the active sites of these molecules in their interactions with the surface of the nanoparticle. The scale bar at the top of this figure demonstrates regions of electron excess (reddish area) to regions of electron deficiency (bluish area) The Hirshfeld point charges and electrostatic potential maps for the optimized structure were obtained using DFT calculations with water as the solvent. The results indicate that the most prominent red areas in the charge distribution maps (Fig. 9) correspond to the active sites of molecules interacting with the nanoparticle. In this case, the atom O4 in caffeic acid, ferulic acid, procyanidin, and vanillic acid; the atom O2 in flavone; the atoms O3 and O4 in chlorogenic acid; the atoms O2 and O4 in epicatechin; and the atoms O6 and O7 in neochlorogenic acid have the highest affinity for interacting with the hydronium jarosite nanoparticle surface.

**Figure 10:**
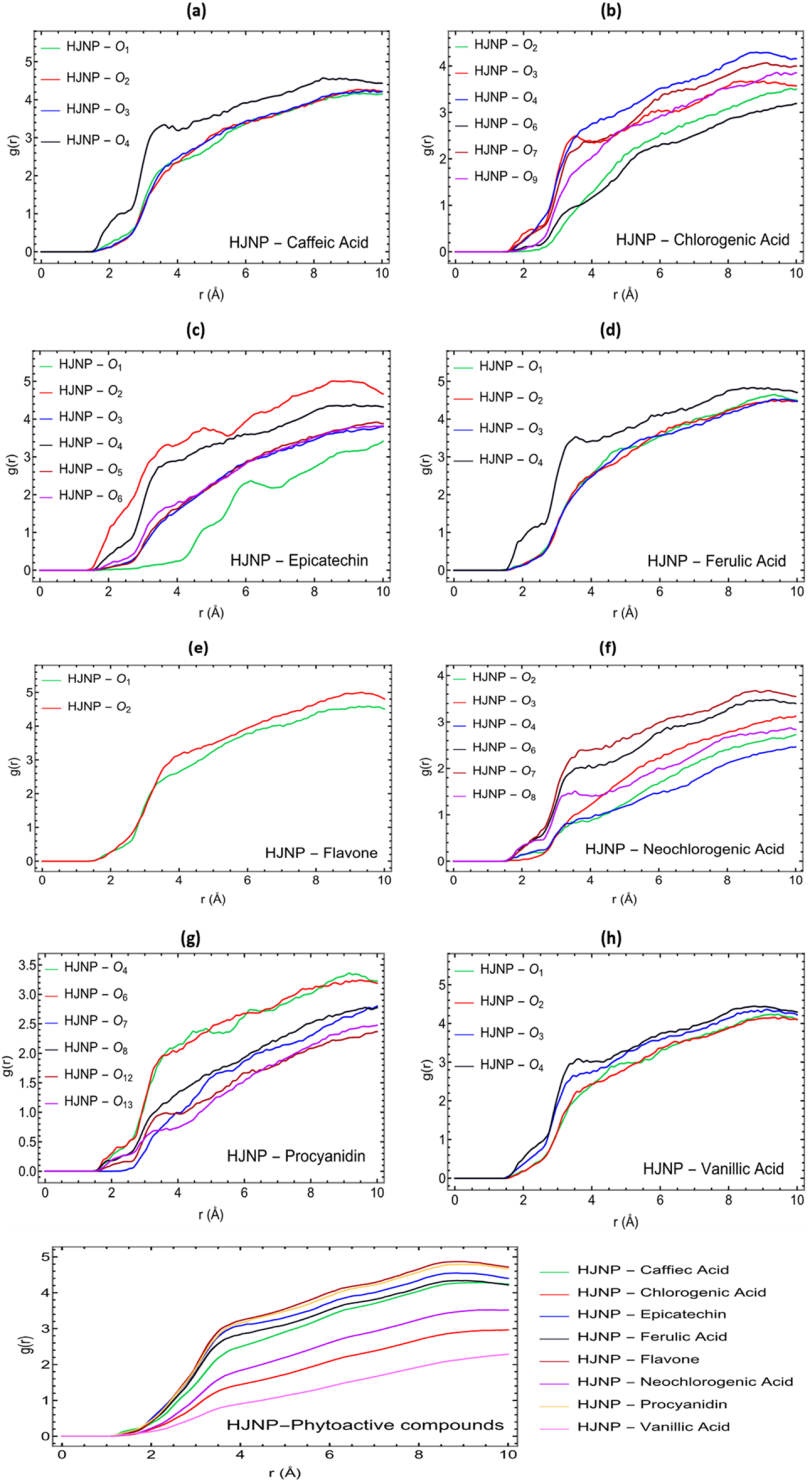
RDF plots for the active sites of (**a**) Caffeic acid, (**b**) Chlorogenic acid, (**c**) Epicatechin, (**d**) Ferulic acid, (**e**) Flavone, (**f**) Neochlorogenic acid, (**g**) Procyanidin, and (**h**) Vanillic acid with respect to the surface of hydronium jarosite nanoparticles. RDF plots of eight phytoactive compounds with respect to the surface of hydronium jarosite nanoparticles (**i**).

The Radial Distribution Function (RDF), g(r), of molecules' active sites with respect to the surface of a spherical hydronium jarosite nanoparticle with a diameter of 2.5 nm is shown in Fig. 10. As illustrated, the g(r) has a maximum peak for the atoms O4 in caffeic acid, ferulic acid, procyanidin, vanillic acid, and chlorogenic acid; O2 in epicatechin and flavone; and O7 in neochlorogenic acid, which exhibit the highest affinity in comparison to other types of oxygen and carbon atoms, which agrees with DFT calculations (Fig. 9). Water solubility is a measure of the amount of active compounds that can dissolve in water at a specific temperature. LogS is directly proportional to an active compound's water solubility and is defined as a standard solubility unit equal to the 10-based logarithm of a molecule's solubility in mol/L. It is reported that logS values greater than − 1 indicate a highly polar molecule, implying higher hydrophilicity and a greater proclivity to interact with water^68^. According to empirical evidence, molecules with log S values between − 1 and − 5 exhibit hydrophilicity, lipophilicity, and aqueous solubility, which enable them to interact with hydrophobic surfaces^68,69^. This section determines logS values for water solubility using the ALOPGPS 2.1 software^70,71^. Flavone has the lowest calculated value of − 4.43, followed by procyanidin, epicatechin, ferulic acid, caffeic acid, neochlorogenic acid, chlorogenic acid, and vanillic acid, which have computed values of − 3.49, − 2.65, − 2.33, − 2.05, − 2.02, − 2.01 and − 1.47, respectively (see Table 1).

**Table 1:** LogS values calculated for phytoactive compounds.

Active compo unds	Caffeic acid	Chloro genic acid	Epicate chin	Ferulic acid	Flavone	Neochl orogenic acid	Procya nidin	Vanillic acid
LogS	− 2.05	− 2.01	− 2.65	− 2.33	− 4.43	− 2.02	− 3.49	− 1.47

The RDF graphs for each phytoactive compound with respect to the surface of the nanoparticle are shown in Fig. 10i. As shown in Fig. 10i, flavone has the highest peak for g(r) and, consequently, the highest density near the nanoparticle’s surface at approximately 3 Å. In addition, as stated earlier, flavone has the lowest value for logS (− 4.43), indicating that it is the least hydrophilic among these active compounds. Together, these two parameters indicate that flavone has a greater affinity to interact with the surface of nanoparticles. Conversely, vanillic acid with the lowest g(r) peak and the highest logS (− 1.47) value has the least affinity to interact with the surface of the nanoparticle. Additionally, the interaction energies (Van der Waals plus electrostatic potential energies) shown in Table 2 reveal that the interaction energy between the active compounds and the nanoparticle is decreasing in the following order: hydronium jarosite nanoparticles—flavone > hydronium jarosite nanoparticles—procyanidin > hydronium jarosite nanoparticles—epicatechin > hydronium jarosite nanoparticles—ferulic acid > hydronium jarosite nanoparticles—caffeic acid > hydronium jarosite nanoparticles—neochlorogenic acid > hydronium jarosite nanoparticles—chlorogenic acid > hydronium jarosite nanoparticles—vanillic acid, while the interaction energy between active compounds and water is decreasing in the following order: water—vanillic acid > water—chlorogenic acid > water—neochlorogenic acid > water—caffeic acid > water—ferulic acid > water—epicatechin > water—procyanidin > water—flavone. The interaction energies (Table 2) and the RDF graphs in Fig. 8 corroborate the discussion on logS values, which indicate that vanillic acid is considerably more hydrophilic (meaning more tendency to interact with water than nanoparticle) than chlorogenic acid, neochlorogenic acid, caffeic acid, ferulic acid, epicatechin, procyanidin, and flavone, respectively.

**Table 2:** Interaction energies extracted from molecular dynamics simulations. HJNP (hydronium jarosite nanoparticles).

Interaction Energies (Van der Waals + electrostatic potential energies)	(kJ/mol)
**HJNP–Caffeic acid**	
HJNP–Caffeic acid	− 234.96 ± 7.2
Caffeic acid–Water	− 2418.15 ± 4.8
**HJNP–Chlorogenic acid**	
HJNP–Chlorogenic acid	− 229.78 ± 4.1
Chlorogenic acid–Water	− 3484.18 ± 4.9
**HJNP–Epicatechin**	
HJNP–Epicatechin	− 287.074 ± 7.2
Epicatechin–Water	− 1803.67 ± 4.1
**HJNP–Ferulic acid**	
HJNP–Ferulic acid	− 261.94 ± 3.3
Ferulic acid–Water	− 1964.09 ± 6
**HJNP–Flavone**	
HJNP–Flavone	− 325.13 ± 5.3
Flavone–Water	− 982.05 ± 4.8
**HJNP–Neochlorogenic acid**	
HJNP–Neochlorogenic acid	− 235.53 ± 6.7
Neochlorogenic acid–Water	− 3447.02 ± 6.2
**HJNP–Procyanidin**	
HJNP–Procyanidin	− 314.5 ± 6
Procyanidin–Water	1505.37 ± 5.5
**HJNP–Vanillic acid**	
HJNP–Vanillic acid	− 201.14 ± 5.2
Vanillic acid–Water	3785.89 ± 4.3

As a preliminary pre-conclusion, one could mention the followings: (i) while the phenolic compound flavone originating from the avocado seed extracts seems driving the formation and the growth of the hydronium jarosite early clusters, (ii) as pointed out previously in section “Morphology and size distribution studies”, the 2-D sheets formation might be driven by an equivalent Frank–van der Merwe layer by layer growth. However, a model of the formation of a very thin precursor film (PF), usually a single molecular layer propagating ahead of the nominal contact line should be explored. This sound hypothesis which deserves to be explored has been demonstrated in several cases where wetting of 2 different fluids gives rise to such a thin precursor film as per the relatively recent investigations of Yuan et al. and Li et al.^72,73^.

## Conclusion

Within this contribution, it is reported for the first time on the bio-engineering of 2-D triangular hydronium jarosite using natural extract of avocado as an effective chelating agent. The molecular dynamics computational modelling showed that the phenolic compound flavone originating from the avocado seed extract, has the highest affinity for interacting with the nanoparticle surface during the phyto-engineering of the hydronium jarosite nanoparticles and the reduction of iron (II) sulphate heptahydrate. XRD and morphological characterization showed that the synthesized hydronium jarosite was representative of crystalline rhombohedral hydronium jarosite in the form of triangular nanosheets decorated on the edges with nanoparticles of approximately 5.4 nm diameter, with EDX elemental profiling confirming the presence of potassium and phosphorus most likely originating from the avocado seed extract. The synthesized nanomaterial further showed diamagnetic behaviour with a high coercivity at a high temperature and low coercivity at low temperatures as well as good electrochemical and electroconductive behaviour, indicating that the phytoengineered hydronium jarosite nanomaterial may find unique utility for electrochemical and diamagnetic applications. Since it has been investigated and reported that the electrochemical and magnetic behaviour of the material is greatly influenced by its morphology, particle size, shape, crystallinity and optical properties^74,75^, future research should focus on using different plants and experimental parameters to optimize the morphological features as well as the electrochemical and magnetic behaviour of hydronium jarosite nanoparticles.

## Data Availability

In line with the journal’s policy and regulations, the datasets used and/or analysed during the current study is available from the corresponding author [Nandipha Botha] on reasonable request.
